# Mesenteric fibromatosis involving the superior mesenteric vein: complex resection and reconstruction in a young female

**DOI:** 10.1093/jscr/rjaf745

**Published:** 2025-10-18

**Authors:** Annita Loizou, Myrto D Keramida, Konstantinos S Giannakopoulos, Nikolaos Machairas, Dimitra Grigoriadou, Georgios C Sotiropoulos, Dimitrios Mantas

**Affiliations:** 2nd Department of Propadeutic Surgery, “Laiko” University Hospital, National and Kapodistrian University of Athens, Ag. Thoma 17 St., Athens 11527, Greece; 2nd Department of Propadeutic Surgery, “Laiko” University Hospital, National and Kapodistrian University of Athens, Ag. Thoma 17 St., Athens 11527, Greece; 2nd Department of Propadeutic Surgery, “Laiko” University Hospital, National and Kapodistrian University of Athens, Ag. Thoma 17 St., Athens 11527, Greece; 2nd Department of Propadeutic Surgery, “Laiko” University Hospital, National and Kapodistrian University of Athens, Ag. Thoma 17 St., Athens 11527, Greece; 1st Department of Pathology, Medical School, Laiko General Hospital, National and Kapodistrian University of Athens, Ag. Thoma 17 St., Athens 11527, Greece; 2nd Department of Propadeutic Surgery, “Laiko” University Hospital, National and Kapodistrian University of Athens, Ag. Thoma 17 St., Athens 11527, Greece; 2nd Department of Propadeutic Surgery, “Laiko” University Hospital, National and Kapodistrian University of Athens, Ag. Thoma 17 St., Athens 11527, Greece

**Keywords:** desmoid tumor, mesenteric fibromatosis, mesenteric neoplasms, vascular surgical procedures, short bowel syndrome (SBS), vein grafting

## Abstract

Primary mesenteric tumors (PMTs) are rare, often presenting asymptomatically until large enough to compress adjacent structures. We present a case of a 36-year-old female with atypical post-cesarean abdominal pain. Imaging revealed an 11 × 10 cm mesenteric mass suggestive of sarcoma or desmoid tumor. Surgical exploration identified a mass involving the mesentery and ileum, necessitating resection of both, along with venous reconstruction of the superior mesenteric vein using a cadaveric graft. A postoperative complication due to venous stasis required a second operation with right colectomy and jejuno-transverse anastomosis. Final pathology confirmed desmoid-type mesenteric fibromatosis. The patient recovered well and remains disease-free and asymptomatic 12 months postoperatively. This case highlights the diagnostic and therapeutic challenges of PMTs, the importance of surgical expertise in vascular reconstruction, and the need for individualized treatment planning to achieve complete tumor resection while minimizing complications such as short bowel syndrome.

## Introduction

Primary mesenteric tumors (PMTs) are rare, comprising a heterogeneous group of lesions with an estimated incidence ranging from 1 in 200 000 to 1 in 350 000 individuals [1]. These tumors may be cystic or solid and exhibit either benign or malignant behavior. Histopathological studies suggest benign tumors are more common [1, 2]. Malignant PMTs, including sarcomas and lymphomas, are exceedingly rare—even less frequent than primary malignancies of the small bowel [2]. Among malignant variants, leiomyosarcomas and fibrosarcomas are notably aggressive. Benign tumors such as lipomas and the rarer leiomyomas are usually asymptomatic unless they attain significant size [1].

Desmoid tumors, also referred to as fibromatosis, represent another rare subset of mesenteric tumors. Although histologically benign, they may behave aggressively at the local level, potentially causing significant morbidity [3].

PMTs often grow slowly but can become large enough to compress adjacent abdominal structures, leading to symptoms such as abdominal pain, bloating, altered bowel habits, anemia due to occult bleeding, or unexplained weight loss [1, 4]. Radical surgical resection remains the treatment of choice when feasible. Tumors discovered incidentally are typically more amenable to resection, whereas symptomatic lesions often require more complex approaches [1, 5].

We herein present the case of a young female referred for surgical management of a large mesenteric tumor.

## Case presentation

**Figure 1:**
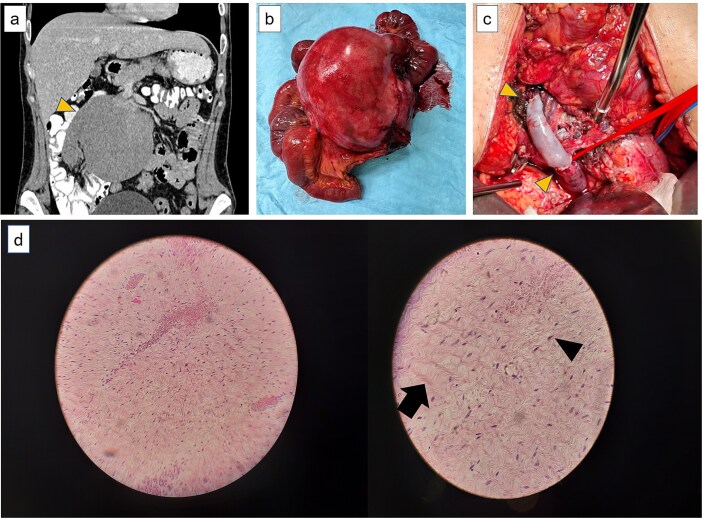
(a) Computer tomography showing the large lesion (arrow) originating from the root of the mesentery in close contact with the superior mesenteric artery and vein. (b) The resected mesenteric lesion along the jejunum and part of the SMV. (c) Vascular reconstruction using a cadaveric graft with anastomoses to the distal and proximal SMV (arrows). (d) Μesenteric fibromatosis of desmoid type (arrow). Spindle-shaped nuclei with a fibroblast-like morphology (arrow head). Low magnification (right)—higher magnification (left).

A 36-year-old Caucasian female with a history of caesarean section 8 months prior presented with atypical abdominal pain and cramping postpartum. Cross-sectional imaging with contrast-enhanced CT and MRI revealed a large, well-defined mesenteric mass measuring 11 × 10 cm. The lesion originated from the mesentery and mesocolon, abutting the small intestine (Fig. 1a). Differential diagnoses included mesenteric sarcoma and desmoid-type fibromatosis. There were no clinical or familial signs suggestive of familial adenomatous polyposis (FAP) or Gardner’s syndrome; thus, genetic testing was not pursued.

Following multidisciplinary team discussion, the patient underwent exploratory laparotomy with curative intent. Intraoperatively, a large mass arising from the mesenteric root and involving a portion of the small bowel—particularly the ileum—was identified. En bloc resection of the tumor, the affected mesentery, and a segment of ileum was performed. The distal portion of the superior mesenteric vein (SMV), traversing the tumor, was ligated and divided (Fig. 1b). End-to-end SMV reconstruction was achieved using a cadaveric venous graft from a compatible donor (Fig. 1c). A terminal jejunostomy was fashioned.

The initial postoperative course was uneventful. On postoperative day 8, bilious output was observed from the abdominal drain. Repeat exploratory laparotomy revealed ischemia of the cecum and terminal ileum due to venous stasis from ileal venous thrombosis. A right hemicolectomy with jejuno-transverse colon anastomosis was performed. The patient recovered well and was discharged in good condition 5 days later. At 12-month follow-up, she remains asymptomatic, with no recurrence or short bowel syndrome (SBS).

Histopathological analysis (Fig. 1d) confirmed desmoid-type fibromatosis (mesenteric fibromatosis) per WHO 2020 classification. The tumor was composed of low-cellularity spindle cells with fibroblastic morphology, without nuclear atypia or mitotic activity. Congested vascular channels and scattered chronic inflammatory infiltrates were noted. No malignant involvement was seen in perienteric lymph nodes. All margins were negative. Immunohistochemistry showed strong nuclear β-catenin positivity. Tumor cells were negative for HHF35, H-caldesmon, desmin, c-KIT, CDK4, MDM2, DOG1, SMA, S-100, and CD34.

## Discussion

Desmoid tumors, also known as mesenteric fibromatosis, are rare, accounting for approximately 0.03% of all neoplasms. Only about 8% arise in the mesentery, most commonly involving the small intestine [4, 6]. These tumors may occur sporadically or with FAP or Gardner’s syndrome. Though benign, they exhibit aggressive local invasion and recurrence, but lack metastatic potential. Risk factors include prior abdominal surgery or trauma, as in this case following a caesarean section [4, 6].

Desmoids often present with vague or absent symptoms and may be found incidentally. Due to slow growth, they are typically asymptomatic early. With progression, they may infiltrate mesenteric structures, including vessels, leading to symptoms such as abdominal pain, altered bowel habits, or bleeding. While imaging may suggest diagnosis, histopathological confirmation is essential [6, 7].

Management ranges from observation to surgery depending on tumor size, symptoms, location, and growth. While conservative treatment suits select patients, surgical resection is preferred for large or symptomatic lesions. In this case, proximity to vital structures necessitated surgical management. Other surgical indications include pain, obstruction, or impaired function [6, 7].

Significant bowel resection may result in SBS, marked by malabsorption, dehydration, and weight loss. Management includes dietary changes, supplementation, and possibly parenteral nutrition [8, 9]. Our patient remains asymptomatic postoperatively.

Complex resections involving desmoid tumors may require vascular resection and reconstruction, particularly when vessels are encased. Preoperative planning is ideal, but intraoperative judgment may dictate en bloc vessel resection to achieve R0 margins. Vascular repair options include synthetic, autologous, or cadaveric grafts. Graft selection depends on availability, involvement extent, and institutional expertise. Surgical experience is vital to managing these intraoperative challenges [2].

## Conclusion

Primary mesenteric tumors, particularly desmoid-type fibromatosis, present diagnostic and therapeutic challenges due to their rarity, locally aggressive behavior, and vascular involvement. In this case, a large mesenteric desmoid tumor mimicked a malignancy and required complex surgical management, including bowel resection and SMV reconstruction with a cadaveric graft, to achieve R0 resection. The favorable outcome highlights the value of individualized treatment planning, a multidisciplinary approach, and technical expertise. Conservative strategies remain appropriate in select cases, but long-term follow-up is essential due to recurrence risk. This case reinforces the importance of considering desmoid tumors in the differential diagnosis of mesenteric masses and demonstrates that complete resection is feasible even in complex scenarios.
